# Entinostat, a histone deacetylase inhibitor, enhances CAR-NK cell anti-tumor activity by sustaining CAR expression

**DOI:** 10.3389/fimmu.2025.1533044

**Published:** 2025-03-07

**Authors:** Dong-Hyeon Jo, Shelby Kaczmarek, Abrar Ul Haq Khan, Jannat Pervin, Diana M. Clark, Suresh Gadde, Lisheng Wang, Scott McComb, Alissa Visram, Seung-Hwan Lee

**Affiliations:** ^1^ Department of Biochemistry, Microbiology, and Immunology, Faculty of Medicine, University of Ottawa, Ottawa, ON, Canada; ^2^ The University of Ottawa Centre for Infection, Immunity, and Inflammation, Faculty of Medicine, University of Ottawa, Ottawa, ON, Canada; ^3^ Department of Cellular and Molecular Medicine, Faculty of Medicine, University of Ottawa, Ottawa, ON, Canada; ^4^ Ottawa Institute of Systems Biology, Faculty of Medicine, University of Ottawa, Ottawa, ON, Canada; ^5^ Human Health Therapeutics Research Centre, National Research Council of Canada, Ottawa, ON, Canada; ^6^ Division of Infectious Diseases, Department of Medicine, Faculty of Medicine, University of Ottawa, Ottawa, ON, Canada; ^7^ Clinical Epidemiology Program, Ottawa Hospital Research Institute, Ottawa, ON, Canada

**Keywords:** natural killer cells, genetic engineering, cryopreservation, chimeric antigen receptor, histone deacetylase inhibitors, entinostat, CD138, multiple myeloma

## Abstract

Allogeneic natural killer (NK) cell therapy has demonstrated significant potential in cancer immunotherapy by harnessing NK cells to target malignancies. CD138-targeting chimeric antigen receptor (CAR)-engineered NK cells offer a promising therapeutic option for multiple myeloma (MM). However, sustaining CAR expression on CAR-NK cells during *ex vivo* expansion poses a challenge to developing effective immunotherapies. In this study, primary NK cells were isolated, cryopreserved, and modified to express anti-CD138 CARs through retroviral transduction. Histone deacetylase inhibitors (HDACi), particularly entinostat (ENT), were applied to enhance CAR expression stability in CAR-NK cells. Our findings indicate that ENT treatment significantly improves and maintains CAR expression, thereby enhancing the cytotoxic activity of CAR-NK cells against CD138-positive multiple myeloma cells. ENT-treated CAR-NK cells exhibited prolonged persistence and more significant tumor reduction in an MM tumor-bearing mouse model, highlighting the therapeutic potential of HDACi-treated CAR-NK cells. This study provides the first evidence that HDAC inhibitors can sustain CAR expression in CAR-NK cells in a promoter-dependent manner, potentially enhancing anti-tumor efficacy in multiple myeloma and underscoring the possible need for further clinical evaluation.

## Introduction

Allogeneic NK cell therapy holds promising potential in cancer immunotherapy by utilizing NK cells from healthy donors to treat patients (1–3). These cells can be readily expanded and stored for “off-the-shelf” use, making the therapy more scalable and accessible (1). NK cells can be engineered with chimeric antigen receptors (CARs) to enhance their cytotoxic effects (4–6). Ongoing research and clinical trials continue to demonstrate the potential of allogeneic CAR-NK therapies, particularly in treating hematological malignancies, where they have shown notable anti-tumor efficacy with a lower risk of complications like graft-versus-host disease (GvHD) and cytokine release syndrome (CRS), which are common in T cell-based therapies (7, 8).

Multiple myeloma (MM) is a clonal plasma cell malignancy characterized by the buildup of malignant plasma cells in the bone marrow, leading to complications such as bone destruction, anemia, and renal impairment (9). Despite advancements in treatments, including proteasome inhibitors, immunomodulatory drugs, CD38-targeting monoclonal antibodies, and more recently, B-cell maturation antigen (BCMA)-targeting CAR-T cells, MM remains largely incurable, with most patients eventually experiencing relapse (10, 11). Targeting CD138 (syndecan-1), a transmembrane proteoglycan highly expressed in myeloma cells and certain epithelial cells, has emerged as a therapeutic strategy, including using a drug-conjugated anti-CD138 antibody (BT026) (12). Additionally, anti-CD138 CAR-T cells have shown promise in preclinical studies, and a phase 1 clinical trial is underway for patients with relapsed and refractory multiple myeloma (Clinical trial: NCT03672318). In NK cell therapy, anti-CD138 CAR has been tested in the NK immortalized cell line, NK92 cells (13). Although on-target, off-tumor effects remain a concern due to CD138 expression in epithelial cells, ongoing optimization in these approaches could lead to significant advances in cell therapies for multiple myeloma, potentially improving survival outcomes for this difficult-to-treat malignancy (14).

Viral transduction-mediated gene delivery is a widely used technique in immune cell engineering to introduce therapeutic genes, such as CARs, into T and NK cells (6, 15). However, one challenge is the potential for transgene silencing after integration into the host genome (16). Gene expression is tightly regulated by histone acetylation, a process controlled by histone acetyltransferases (HATs) and histone deacetylases (HDACs) (17). High levels of histone acetylation result in a more open chromatin structure, improving RNA polymerases accessibility and facilitating gene expression (17). Recently, HDAC inhibitors (HDACi) have increased histone acetylation, resulting in more relaxed chromatin and enhanced promoter accessibility (17, 18). HDAC inhibitors like entinostat (ENT) and valproic acid (VPA) have shown promise in increasing transgene expression in cancer cell lines (19–21). In T cells, T cell receptor (TCR) engineered cells have demonstrated enhanced expression following HDACi treatment (22). While TCR-T cells have demonstrated HDACi-mediated transgene overexpression, it is noteworthy that prior studies involving anti-CD19 CAR-T cells treated with HDAC inhibitors, such as panobinostat and entinostat, did not show enhanced CAR expression (23). This discrepancy may be linked to differences in promoter responsiveness to HDACis, as demonstrated by studies on adenoviral vectors containing cytomegalovirus (CMV) and PPT promoters (24). In these studies, the CMV promoter exhibited responsiveness to HDACis, resulting in GFP overexpression, whereas the PPT promoter showed no such effect.

In this study, we investigated the anti-tumor activity of anti-CD138 CAR-engineered NK cells against multiple myeloma, including the option for cryopreservation. After the CAR transduction of NK cells, we observed a progressive decrease in CAR expression in CAR-NK cells during *ex vivo* expansion. Notably, HDACi treatment was able to restore CAR expression, thereby enhancing effector function both *in vitro* and *in vivo*. To our knowledge, this is the first report to assess the impact of HDACi treatment on transgene overexpression in CAR-NK cells in a promoter-dependent manner. Overall, this study highlights the potential of HDACi treatment to enhance the efficacy of CAR-NK cell therapy by upregulate CAR expression.

## Methods

### 1. Cancer cell line culture

Multiple myeloma cell lines, MM1.R and MM1.S, as well as B lymphoma cell lines, Ramos and Raji, were obtained from ATCC and cultured in RPMI-1640 medium (350-000-CL; Wisent) supplemented with 10% heat-inactivated fetal bovine serum (HI-FBS) (12484028; Gibco), 100U/mL Penicillin and 100µg/mL Streptomycin (Pen/Strep) (SV30010; HyClone), 55µM β-Mercaptoethanol (21985023; Gibco), and 20mM HEPES (CA12001-708; VWR) (hereafter called RP10 medium). MDA-MB-231 cells were also obtained from ATCC, while Lenti-X 293T cells were purchased from Takara (632180), and both were cultured in high-glucose DMEM (319-005-CL; Wisent) supplemented with 10% HI-FBS and 100µg/mL Pen/Strep. NK92 cells were cultured in RP10 medium supplemented with 200U/mL human recombinant IL-2 (NCI Preclinical Repository, USA). Two different MM1.S-firefly luciferase (FLUC) cells, MM1.S-FLUC-red fluorescent protein (RFP) and MM1.S-FLUC-blasticidin cells, were utilized for *in vivo* experiments.

### 2. Generation of master stocks of human primary NK cells

The Ottawa Health Science Network Research Ethics Board (#20200527-01H) and the University of Ottawa (#H-01-21-6568) authorized the collection of whole blood from healthy adults. Peripheral blood mononuclear cells (PBMCs) were extracted using Ficoll (45001750; Cytiva) gradient centrifugation, and NK cells were isolated as previously demonstrated (4). The isolated primary NK (pNK) cells were immediately cultured with two times the number of irradiated K562 feeder cells, expressing membrane-bound IL-21 and 4-1BBL (a kind gift from CYTOSEN), along with 100U/mL of IL-2 in RP-10 medium for three days (25). After three days, half of the medium was replaced with NKMACS medium (130-114-429; Miltenyi Biotech), and the cells were expanded for two additional days. On day five, an equal volume of NKMACS medium was added. By day six or seven, the partially expanded pNK cells were frozen at -80°C using a freezing medium containing 90% FBS and 10% DMSO (BP231-100; FisherBioReagents).

### 3 Molecular cloning

To generate a retroviral pMIG-Green fluorescent protein (GFP)-IL-15 plasmid, EcoRI-GFP-IL-15-PacI fragments were produced by digestion with EcoRI (FD0274; Thermo Fisher Scientific) and PacI (FD2204; Thermo Fisher Scientific). The resulting fragment was cloned into an EcoRI- and PacI-treated pMIG plasmid (gifted by William Hahn; 9044; Addgene). To create the pMIG-GFP-IL-15-EFS-CAR construct, the FMC63-28Z CAR plasmid was obtained from Addgene (#135991, deposited by Scott McComb; http://n2t.net/addgene:135991; RRID: Addgene_135991) (26). The anti-CD138 single-chain variable fragment (scFv) gene fragment, purchased from GeneArt String (Thermo Fisher Scientific), was cloned into the FMC63-28Z CAR by replacing FMC63 with anti-CD138 scFv using BpiI-Golden Gate Cloning, as described in previous studies (FD1014; Thermo Fisher Scientific) (26). The entire construct, including the EFS promoter and anti-CD138 CAR, was amplified by PCR and cloned into the pMIG-GFP-IL-15 plasmid using PacI and SalI (FD0644; Thermo Fisher Scientific) restriction sites. For the generation of different promoter-containing CAR constructs, the CMV promoter was extracted from the pLenti-CMV-GFP-SV40-puro plasmid using CMV-F (AGTCGTACCGGTCGTTACATAACTTACGGTAA) and CMV-R (AACATCGGATCCCGCGTCACGACACAGCTCTGCTTATATAGACCT) primers and the MSCV promoter (MSCV-F: AGTCGTACCGGTAATGAAAGACCCCACCTGTA and MSCV-R: AACATCGGATCCCGCGTCACGACACGGCGCGCCGAGTGAGGGGTT) was transferred from the pMIG-GFP-IL-15 plasmid to a QC5-EFS-anti-Epidermal growth factor receptor (EGFR) CAR-IL-15 construct by replacing the EFS promoter using BshTI and BamHI. To generate CD138-positive Ramos cells, the SV40-puro from the pLenti-CMV-GFP-SV40-puro plasmid was replaced with an EFS-multiple cloning site (MCS) using MluI (FD0564; Thermo Fisher Scientific) and Eco91I (FD0394; Thermo Fisher Scientific), with the EFS-MCS fragment purchased from Thermo Fisher Scientific (GeneArt String). The CD138 fragment (GeneArt String, Thermo Fisher Scientific) was then inserted into the pLenti-CMV-GFP-EFS-MCS plasmid using Bsp119I (FD0124; Thermo Fisher Scientific) and NheI (FD0974; Thermo Fisher Scientific). PCR was performed using Platinum™ SuperFi™ PCR Master Mix (#12358050; Thermo Fisher Scientific). All cloning was confirmed by sequencing conducted at the Ottawa Hospital Research Institute (OHRI) DNA sequencing facility, and sequencing results were analyzed using SnapGene (Dotmatics).

### 4 Retroviral and lentiviral vector production

To produce retroviral vectors, we followed a previously published method with slight modifications (4). A total of 1.2 × 10^6^ Lenti-X 293T cells were plated per well on a 6-well plate (83.3920, Sarstedt) in 2 mL of serum-containing Opti-MEM medium (51985091, Gibco). The next day, the cells were transfected with 1,200ng of retroviral transgene plasmids, 1,200ng of MLV-gag-pol, 500ng of pMD2.G (deposited by Didier Trono, Addgene #12259; http://n2t.net/addgene:12259; RRID: Addgene_12259), and 100ng of BaEV-TR (27) using Lipofectamine 3000 (L3000015, Invitrogen) (6µL P3000 and 7µL Lipofectamine 3000). After a four-hour incubation, the medium was completely removed and replaced with 2 mL of fresh Opti-MEM medium. The first viral supernatant was collected the day after transfection, followed by a fresh medium replacement for the second harvest on day 2. Viral supernatants were filtered using a low protein binding PES filter (83.1826, Sarstedt) and stored at -80°C. To produce lentiviral vectors and the functional titer calculation, we followed the method previously published (4).

### 5 Primary NK cell transduction and expansion

Cryopreserved primary NK (pNK) cells of master stocks were thawed and rested overnight in a DS medium (28), containing 10% heat-inactivated fetal bovine serum (HI-FBS) and 100U/mL IL-2. The following day, the pNK cells were transduced in a flat-bottom plate pre-coated with 20µg/mL Retronectin (T100B, Takara Bio). Viral supernatant and 100U/mL IL-2 were added to the plate. The cells were centrifuged at 1,000g for 30 minutes at 32°C and cultured overnight at 37°C and 5% CO2. The next day, the engineered pNK cells were stimulated with irradiated feeder cells at a 1:5 ratio and 100U/mL IL-2. The pNK cells were then expanded, with the culture medium being replaced every 2 to 3 days with a fresh DS medium containing 100U/mL IL-2. For experiments involving HDAC inhibitor (HDACi) treatment, NKMACS medium containing 10% HI-FBS and gentamicin (10µg/mL, 15710-064, Gibco) was used.

### 7 Immunostaining

Antibody-based cell immunostaining was performed as previously described (4) with the following antibodies, reagents, and beads. Data were analyzed by Kaluza Analysis 2.1 (Beckman Coulter) and Flowjo v10.8.1 (BD Life Sciences). Jackson ImmunoResearch Laboratories Inc: anti-human Fab(ab’)_2_ (109-605-006, AF647), anti-Alpaca’s VHH (128-605-230, AF647). Invitrogen™: NKp46 (53-3359-42, AF488), CTLA4 (11-1529-42, FITC), NKp30 (12-3379-42, PE), NKG2D (46-5878-42, PerCP-eFluor710), CD16 (46-0118-42, PerCP-eFluor710), IgG1k (46-4714-82, PerCP-eFluor710), LAG3 (56-2239-41, APC), CD38 (67-0389-42, CD702). BD Biosciences: CD57 (560844, PE). Biolegend: IgG2k (400506, FITC), KIR2DL2/3 (312604, FITC), NKp44 (325108, PE), NKG2C (375003, PE), murine CD45 (110708, PE), IgG1k (400112, PE), TNF-α (557068, PE),CD138 (356514, PE/Cyanine7), IgG1k (400125, PE/Cyanine7), CD107a (328620, APC), NKG2A (375108, APC), KIR3DL1 (312716, APC), IgG1k (400120, APC), IFN-γ (502548, APC/Fire710), CD56 (318328, BV421), CD3 (317332, BV510), CD138 (356520, BV605), IgG1k (562652, BV605), human CD45 (304048, BV785), IFN-γ (502542, BV785). LIVE/DEAD™ Fixable Viability dyes: Yellow (L34959, Invitrogen), Aqua (L34957, Invitrogen), Near-IR (L34976, Invitrogen), Green (L23101, Invitrogen). UltraComp eBeads™ Plus Compensation Beads (01-3333-42, Invitrogen).

### 8 Puromycin staining

Puromycin staining was performed based on the previously published paper (29). Briefly, NK cells were plated on a 48-well plate (83.3923, Sarstedt) with or without 100mM 2-Deoxy-D-Glucose (2DG) (D8375-5G, MilliporeSigma) and 1µM oligomycin (O4876-5MG, MilliporeSigma). Cells were treated for 45 minutes at 37 °C. Then, the cells received 10µg/ml puromycin (450-162-XL, Wisent) and incubated for 45 minutes at 37 °C. The cells were washed with ice-cold PBS and stained with a live-dead dye and an anti-puromycin antibody (381508, AF647, Biolegend). A Cyto-Fast™ Fix/Perm Buffer Set (426803, Biolegend) was used for the intracellular puromycin staining by following the manufacturer’s instructions.

### 9 *In vitro* CD107a, IFN-γ, and TNF-α assays

For CD107a, IFN-γ, and TNF-α assays, effector and target cells were plated at a 1:1 ratio on a 96-well plate, with an anti-CD107a antibody (if required) added to each well. The plate was incubated at 37°C and 5% CO2. After one hour of incubation, brefeldin A (00-4506-51; Invitrogen) was added to the culture. NK cells and target cells were co-incubated for an additional three hours. Following the incubation, the cells were washed with phosphate-buffered saline (PBS) (10010049, Gibco) containing 2% HI-FBS and stained with appropriate antibodies for flow cytometric analysis.

### 10 *In vitro* killing assay

For short-term killing assay, target cells were labeled with cell-trace violet (CTV) dye (C34571, Invitrogen™) according to the manufacturer’s instructions. The CTV-labeled target cells were then co-cultured with NK cells for four hours at varying effector-to-target cell ratios. The percentage of dead cells within the CTV-positive target cell population was subsequently analyzed by flow cytometry.

For long-term killing assays, firefly luciferase (FLUC) expressing target cells were used based on the previously published studies (30). Primary NK cells were co-cultured overnight with FLUC-expressing target cells at varying effector-to-target ratios in a 96-well assay plate (31113, Labselect). Following incubation, D-luciferin (122799-5, Revvity) was added to the wells to reach a final 150 µg/mL concentration. Luminescence was measured using the Synergy H1 Multi-Mode Plate Reader (Biotek) to assess the cytotoxic activity of the pNK cells against the target cells. Cytotoxicity was calculated based on the equation below. (Lum: Luminescence)


$$(1-\frac{Lum^{\text{Target}+\text{NK cells}}}{Lum^{\text{Target alone}}-Lum^{\text{medium}}})\times 100$$

### 11 Histone deacetylase inhibitor treatment

Entinostat (A8171-5.1, Labclinics), Valproic acid (P4543-10G, MilliporeSigma), and RGFP966 (16917-1, Cayman Chemical) were obtained commercially. For short-term exposure and dosage evaluation, overnight rested pNK cells were transduced with CAR-IL-15 retroviral vectors at a multiplicity of infection (MOI) of 3, following the standard pNK cell transduction protocol. The transduced cells were stimulated and expanded for five days (TD + 5). After five days, the modified NK cells were cryopreserved at -80°C using a freezing medium. The engineered and cryopreserved NK cells were later thawed and rested overnight for HDAC inhibitor (HDACi) titration. The resting NK cells were treated with varying concentrations of HDACis (as indicated in the figures), incubated for two days, and then immunostained to validate transgene expression.

For long-term exposure, pNK cells were transduced, stimulated, and expanded for five days. Afterward, the cells were divided into two groups: cultured in an HDACi-containing medium and a DMSO-containing (non-treated, NT) medium. The medium was refreshed every two to three days. Prior to *in vitro* and *in vivo* experiments, ENT-treated cells were washed once with NKMACS medium.

### 12 *In vivo* tumor control experiments

A breeding pair of NOD.Cg-Prkdcscid Il2rgtm1Wjl/SzJ (NSG) mice were acquired from Jackson Laboratories, and the colony was housed in the specific pathogen-free animal facility at the University of Ottawa, adhering to Canadian Council on Animal Care guidelines. Male NSG mice (8 to 12 weeks old) received 2.5 × 10^6^ MM1.S cells expressing firefly luciferase intravenously. The mice were then assigned to groups (n=5/groups treated with NK cells or n=2 or 3 for the PBS control group). Three or seven days post-injection of the target cells, the mice were administered 1 × 10^7^ pNK cells intravenously (for the HDACis study, twice at six-day intervals). The luciferase signal was monitored using the IVIS^®^ Spectrum (Perkin Elmer) and Newton animal imaging system (VILBER; in the middle of this project, Newton was purchased). Blood samples were collected from the saphenous vein. All procedures were approved by and conducted in compliance with the animal care guidelines of the University of Ottawa.

### 13 Statistical analysis

The mean values for multiple factors were analyzed using two-way ANOVA, comparing the means of the groups. A t-test was used for two-factor comparisons to evaluate the mean values performed in GraphPad Prism 9 (Dotmatics). Statistical significance was defined as p < 0.05. Data are presented as mean ± SD, and all graphs were generated and analyzed using GraphPad Prism 9.

## Results

### NK cells from a cryopreserved master stock are expandable *ex vivo* and suitable for CAR engineering

**Figure 1:**
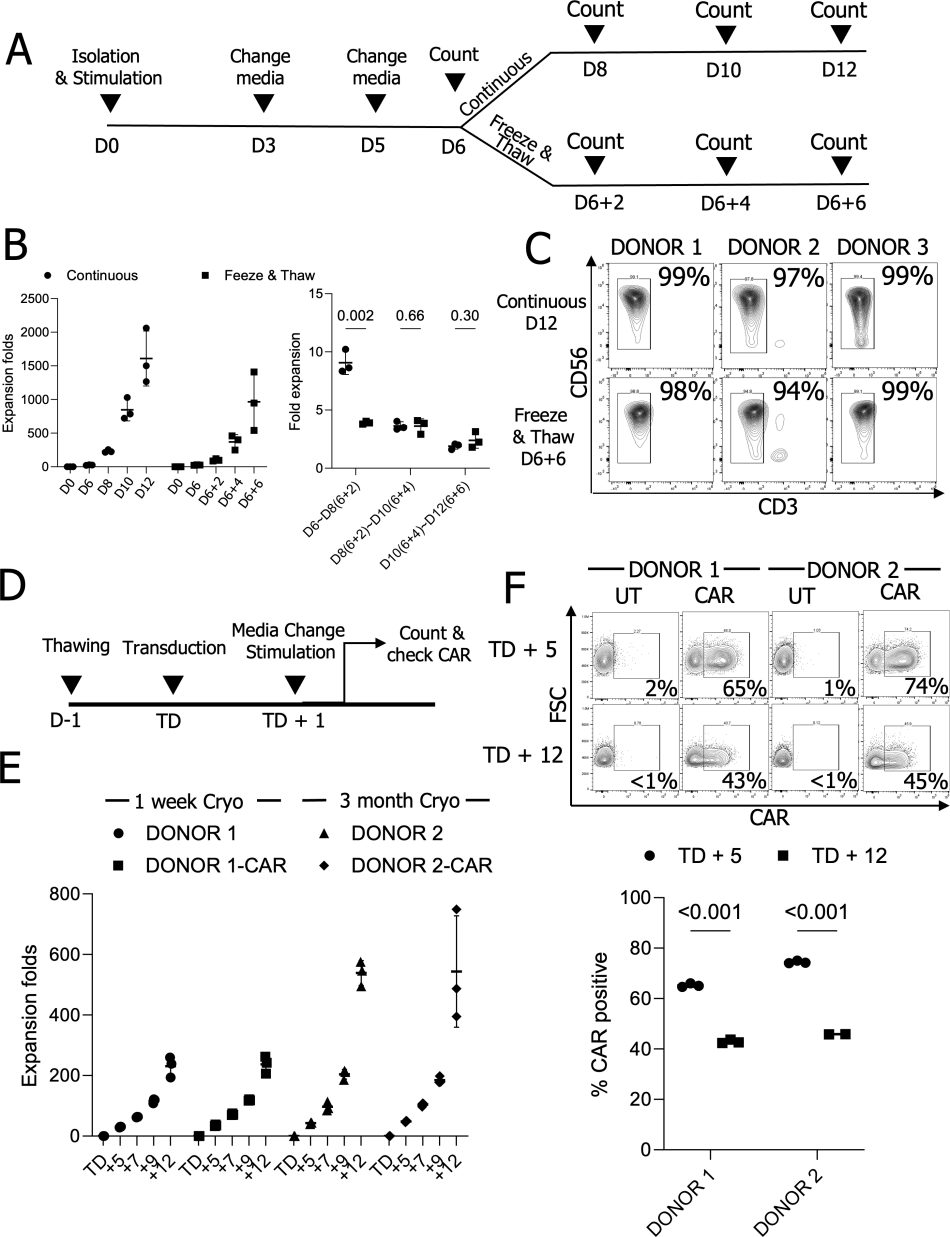
Cryopreserved master stock NK cells can be expanded and engineered for CAR therapy. **(A)** The schematic diagram for expansion of cryopreserved NK cells. **(B)** NK cell expansion folds at two-day intervals (n=3 donors’ NK cells) **(C)** NK cell purity analysis from expanded NK cells (n=3 donors’ NK cells). **(D)** The timeline for NK cell engineering and expansion. **(E)** Expansion folds in UT and chimeric antigen receptor (CAR) engineered NK cells. (n=2 donors’ NK cells with triplicate transduction) **(F)** CAR expression on days 5 and 12 after the transduction (TD + 5 and TD + 12). (n=2 donors’ NK cells with triplicate transduction); UT, Untransduced; Cryo, Cryopreserved NK cells.

Traditional NK cell engineering involves a continuous process of NK cell isolation, expansion, engineering, and further expansion, where the efficiency of NK cells expansion and engineering is highly donor-dependent. To streamline this process and minimize redundant NK cell isolations, we hypothesized that cryopreserving NK cells at the early stages of expansion could improve NK cell readiness for CAR-NK cell production. pNK cells from three donors were expanded using irradiated K562 cells expressing 4-1BBL and membrane-bound (mb) interleukin (IL)-21 in the presence of IL-2 (25). After 6 days of expansion, a master stock was cryopreserved in FBS containing 10% DMSO. After 2 days of cryopreservation, a vial of the master stock was thawed, and the pNK cells were further expanded (**Figure 1A**). Their expansion rates were slightly lower than those of pNK cells without cryopreservation. pNK cells that had not been frozen achieved an average 1,609-fold expansion over 12 days. pNK cells from the frozen master stock showed a transient delay in expansion but eventually reached similar growth rates to those of unfrozen pNK cells, achieving an average 846-fold expansion (D6 + 6). Although the total expansion was 1.9-fold lower after 12 days, the expansion rates at two-day intervals following cryopreservation were comparable (**Figure 1B**). Phenotypes of the expanded cells were similar and showed high purity (**Figure 1C**, **Supplementary Figure S1**).

To explore the potential for engineering pNK cells from a master stock, we generated lentiviral vectors encoding 4-1BB and CD3ζ signaling-based chimeric antigen receptors (CARs), along with constitutive expression of human IL-15. We thawed pNK cells from master stocks that had been cryopreserved for one week or three months in a DS medium (28) containing 100 U/mL IL-2 and rested them overnight to remove damaged cells. The following day, 5 × 10^4^ pNK cells were transduced with lentiviral vectors at a MOI of 2. After overnight transduction, the viral supernatant was replaced with fresh medium containing 2.5 × 10^5^ (5×) irradiated feeder cells (**Figure 1D**). The medium was changed every two days, and expansion rates and CAR expressions were assessed five days post-transduction (TD + 5) and up to 12 days (TD + 12). pNK expansion from cells cryopreserved for either one week or three months was comparable for both unmodified and CAR-engineered pNK cells (**Figure 1E**). CAR expression in CAR-transduced NK cells decreased similarly over time in NK cells cryopreserved for one week or three months when measured on TD + 5 and TD + 12 (**Figure 1F**). Taken together, primary NK cells from a cryopreserved master stock are expandable *ex vivo* and are suitable for engineering via viral transduction.

### Anti-CD138 CAR enhances NK cell function *in vitro* and *in vivo*; however, CAR expression decreases during expansion *ex vivo*

**Figure 2:**
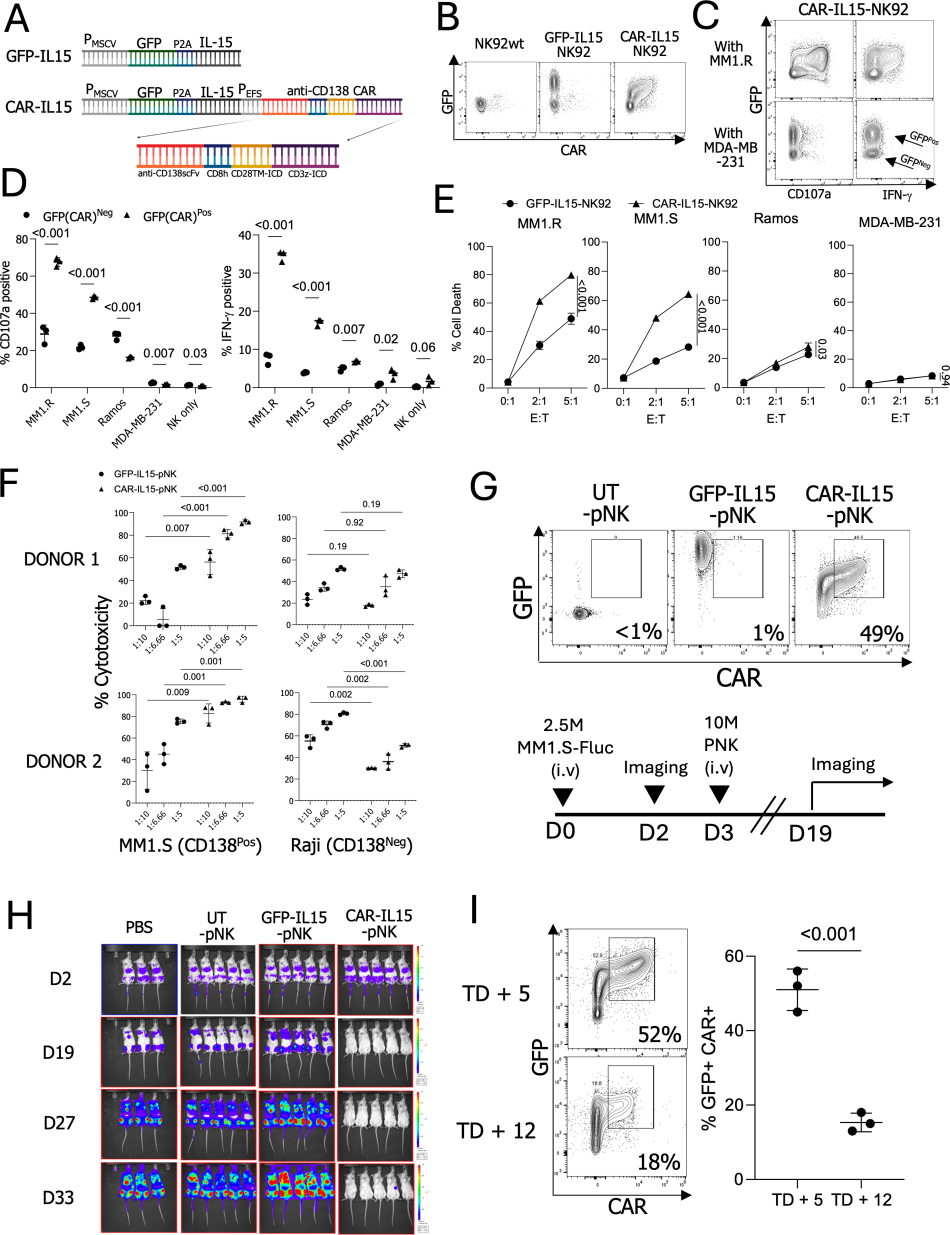
Functionality and CAR expression of anti-CD138-CAR engineered NK cells. **(A)** Design of the anti-CD138 CAR construct. **(B)** GFP and CAR expressions in engineered NK92 cells. **(C)** The CD107a and IFN-γ-expression patterns in modified NK92 cells co-cultured with CD138-positive target MM1.R and CD138-negative target MDA-MB-231 cells. **(D)** CD107a and IFN-γ expressions in NK92 cells with or without target cells. **(E)** NK92 cell killing assay with CD138 positive and negative target cells **(F)** Killing assay using engineered pNK cells with MM1.S (CD138-positive, CD138^Pos^) and Raji (CD138-negative, CD138^Neg^) cells. (n=2 donors’ NK cells) **(G)** Injected NK cell GFP and CAR expressions and timeline of the performed *in vivo* experiment. **(H)** *In vivo* imaging. Signals indicate firefly luciferase-expressing MM1.S cells. **(I)** CAR expressions in NK cells on TD + 5 and TD + 12 (n=3 cryopreserved donors’ NK cells).

To enhance the ability of expanded NK cells to kill myeloma cells, we designed an anti-CD138 CAR using a BpiI-based type II restriction enzyme cloning approach (26). This CAR construct includes a CD8 hinge, a CD28 transmembrane domain, and CD28-CD3ζ intracellular signaling domains in a lentiviral vector. The anti-CD138-CAR sequence was integrated into the retroviral vector pMIG-GFP-IL-15 to generate a retroviral CAR transgene plasmid (**Figure 2A**). This transfer was done due to the use of the GFP-IL-15 backbone. NK92 cells were transduced with retroviral vectors containing GFP-IL-15 and GFP-IL-15-CAR (CAR-IL-15) constructs, and surface CAR expression was assessed using an anti-human Fab(ab’)2 antibody (31). NK92 cells transduced with GFP-IL-15 showed 71% GFP expression with minimal background anti-Fab(ab’)2 signals, whereas CAR-IL-15-NK92 cells displayed 69% GFP expression and over 50% CAR-positive populations (**Figure 2B**).

To assess NK cell functionality and cytotoxicity, GFP-IL-15-NK92 and CAR-IL-15-NK92 cells were co-cultured with CD138-positive multiple myeloma cell lines (MM1.R and MM1.S) and CD138-negative cell lines (Ramos and MDA-MB-231) (**Supplementary Figure S2A**). CD138-positive targets, MM1.R and MM1.S, induced an average of 67% and 48% CD107a expression and 34% and 17% IFN-γ production in CAR-IL-15-NK92 cells, respectively, compared to 28% and 21% CD107a and 7% and 3% IFN-γ in untransduced (UT) NK92 cells. In contrast, negative control targets (Ramos, MDA-MB-231), or NK cells alone, did not enhance NK cell responses in CAR-IL-15-NK92 cells beyond those seen in UT-NK92 cells (**Figures 2C, D**). CAR-IL-15-NK92 cells demonstrated enhanced cytotoxicity against MM1.R and MM1.S compared to GFP-IL-15-NK92 cells, while cytotoxicity against Ramos and MDA-MB-231 cells was limited, regardless of CAR expression (**Figure 2E**). These results underscore the specificity and efficacy of anti-CD138 CAR-engineered NK cells in selectively targeting multiple myeloma cells.

To evaluate the functionality of the anti-CD138 CAR in expanded pNK cells derived from cryopreserved stocks, we thawed frozen pNK cells from master stocks of three different donors, genetically modified the pNK cells with retroviral vectors encoding either GFP-IL-15 or CAR-IL-15, and expanded them for 5 days. All three CAR-IL-15-pNK cell cultures exhibited positive staining for anti-CAR, while GFP-IL-15-pNK and UT-pNK cells showed no CAR signals. Both GFP-IL-15 and CAR-IL-15-pNK cells exhibited GFP-positive signals (**Supplementary Figure S2B**). Next, we assessed the anti-tumor activity of CAR-IL-15-pNK cells. pNK cell cytotoxicity was evaluated against CD138-positive MM1.S cells and CD138-negative Raji cells. Notably, CAR-IL-15-pNK cells from two donors exhibited significantly higher cytotoxicity against CD138-positive MM1.S but not CD138-negative Raji cells (**Figure 2F**). To address CAR specificity, we generated Ramos cells expressing CD138 (Ramos-CD138). Ramos-CD138 cells induced stronger CD107a and IFN-γ signals than Ramos cells (**Supplementary Figure S2C**). We also evaluated the *in vivo* cytotoxicity of CAR-IL-15-pNK cells by injecting 2.5 × 10^6^ MM1.S cells into NSG mice, followed three days later by 1 × 10^7^ pNK cells (**Figure 2G**). In line with our *in vitro* findings, CAR-IL-15-pNK cells demonstrated significantly enhanced eradication of multiple myeloma cells compared to GFP-IL-15-pNK and UT-pNK controls (**Figure 2H**). Although our anti-CD138 CAR construct improved the anti-tumor function with 49% CAR+ pNK cells *in vivo*, we also observed a marked decline in CAR expression during *ex vivo* expansion, with expression dropping from 51% on TD + 5 to 15% on TD + 12, leading to the limited use of CAR-pNK cells within short expansion time (**Figure 2I**).

### Histone deacetylase inhibitors enhance GFP and CAR expression in NK cells in a dose- and promoter-dependent manner

**Figure 3:**
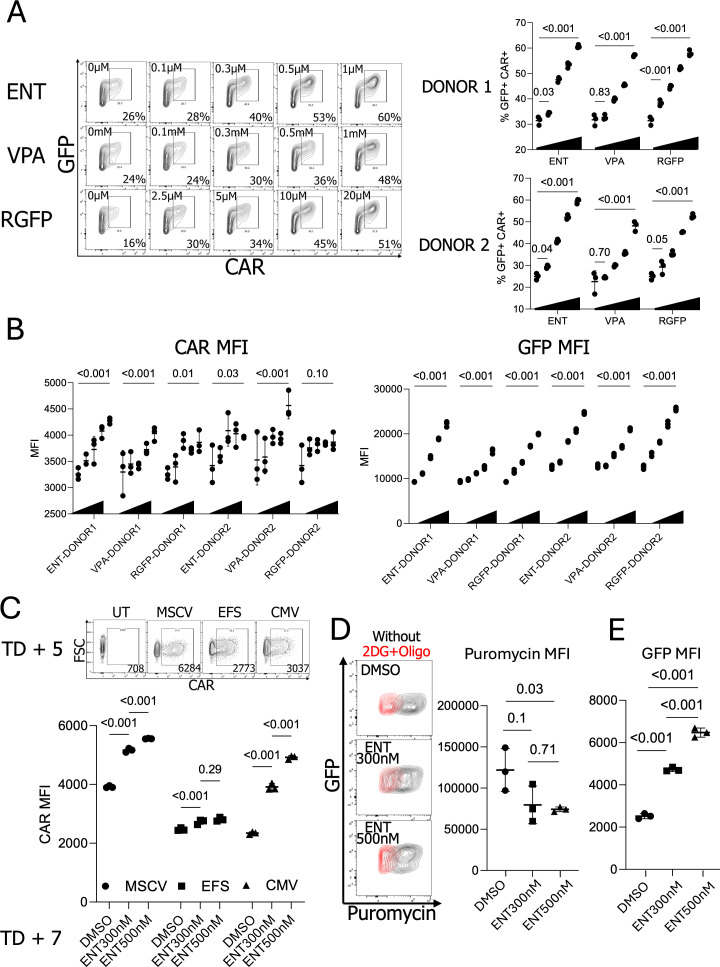
Enhanced CAR expression in NK cells with the treatment of histone deacetylase inhibitors. **(A)** Effects of different concentrations of Histone deacetylase inhibitor (HDACi) in GFP+ CAR+ population. (n=2 donors’ NK cells) **(B)** CAR and GFP MFIs in HDACi-treated pNK cells. (n=2 donors’ NK cells) **(C)** ENT 300nM and 500nM-treated MSCV, EFS, and EFS-CAR-engineered pNK cells. (n=1 donor’s NK cells with triplicate transduction) **(D)** Puromycin-based NK cell translation capacity confirmation. Puromycin staining was performed with or without metabolic inhibitors, 2DG, and oligomycin. (n=1 donor’s NK cells) **(E)** GFP expressions in the cells tested for the puromycin staining; ENT, Entinostat; VPA, Valproic acid; RGFP, RGFP966; MSCV, Murine stem cell virus promoter; EFS, EF1-α small promoter; CMV, Cytomegalovirus promoter; 2DG, 2-Deoxy-D-glucose.

To explore whether this CAR downmodulation can be overcome by adding small molecule inhibitors targeting epigenetic machinery, we employed histone deacetylase inhibitors (HDACi). Specifically, we chose to use Entinostat (ENT, an HDAC class I and IV inhibitor) (32), Valproic Acid (VPA, an HDAC class I and IIa inhibitor) (33), and RGFP966 (RGFP, an HDAC 3 inhibitor) (34) since ENT and VPA previously showed improved transgene regulations (19–21). For consistency, we cryopreserved anti-CD138 CAR-expressing pNK cells on TD + 5, then thawed, rested overnight, and cultured them with HDAC inhibitors for 2 days. Remarkably, all HDAC inhibitors tested significantly increased the GFP+ CAR+ populations in a dose-dependent manner. In the absence of HDAC inhibitors, the GFP+ CAR+ NK cell population averaged 31.6%. However, this population increased to 60.6% with ENT, 57.1% with VPA, and 57.9% with RGFP966 at the highest concentrations tested, without significant toxicity (**Figure 3A**, **Supplementary Figure S3**). When the mean fluorescence intensity (MFI) of CAR and GFP was assessed, CAR expression generally improved with HDACi treatment; however, the treatment had a stronger effect on GFP expression (**Figure 3B**). Given that GFP and CAR expression are controlled by two distinct promoters in the GFP-CAR-IL-15 construct, we hypothesize differential effects of HDAC inhibitors on these promoters. Since all three HDAC inhibitors produced similar effects in NK cells, we primarily used ENT in subsequent experiments.

To determine if ENT exert different effects on various promoters, we generated lentiviral vectors containing an unrelated anti-EGFR CAR construct (35) driven by three distinct promoters: The murine stem cell virus (MSCV) promoter, EF1a small (EFS) promoter, and Cytomegalovirus (CMV) promoter. Since the MSCV and CMV promoters have shown improved expressions after HDACi treatment (19, 36), we anticipated that HDAC inhibitors in NK cells would significantly enhance MSCV and CMV promoter activities. Consistent with our hypothesis, ENT significantly boosted CAR expression driven by both the MSCV and CMV promoters but had a minimal effect on the EFS promoter. Interestingly, after ENT treatment, CAR expression driven by the CMV promoter exhibited a higher MFI than the EFS promoter despite lower baseline expression in the absence of HDACi (**Figure 3C**). Interestingly, we observed enhanced CAR expression in the treatment of ENT, even though the CAR expression is under the EFS promoter in our retroviral CAR construct. Currently, we reason that the leaky upstream MSCV promoter influences the downstream EFS promoter, as reported (37, 38). To determine whether the increased GFP+ CAR+ population resulted from enhanced protein translation, we treated NK cells with puromycin and measured puromycin signals using flow cytometry as a surrogate marker for actively translating polypeptides (29). Puromycin staining revealed that ENT reduced protein translation in pNK cells (**Figure 3D**). Despite reduced translation, GFP expression was still higher in the presence of ENT, indicating that ENT-mediated transgene overexpression is not linked to general protein synthesis in NK cells (**Figure 3E**). Taken together, histone deacetylase inhibitors enhance CAR expression in NK cells in a promoter-specific manner.

### Entinostat treatment reduces the background of NK cell degranulation but does not affect CAR-mediated NK cell degranulation

**Figure 4:**
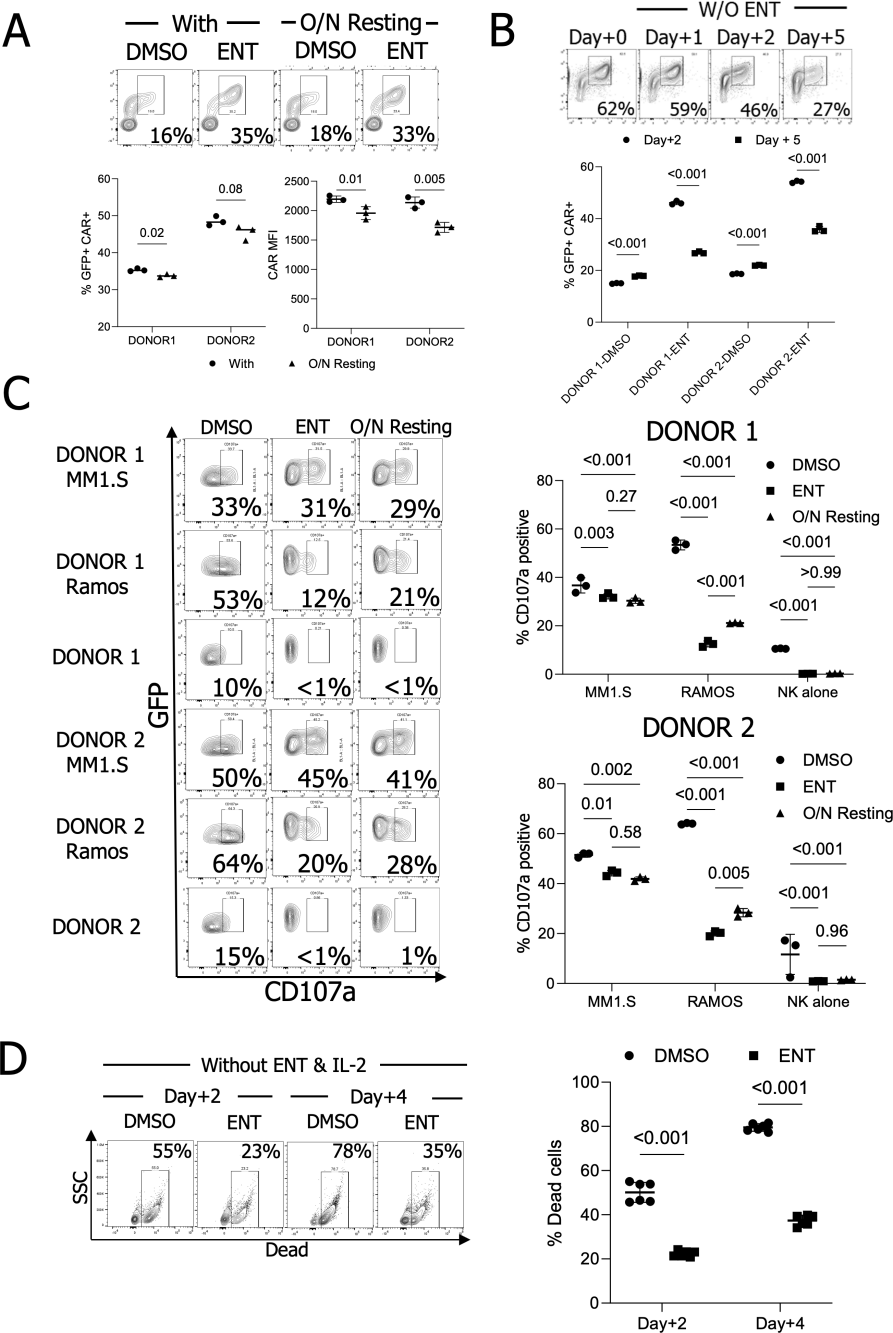
CAR maintenance, degranulation, and survival of ENT-treated NK cells *in vitro*. **(A)** GFP+ CAR+ population and CAR MFI analysis after overnight resting. (n=2 donors’ ENT 500nM-treated NK cells) **(B)** GFP+ CAR+ population maintenance analysis for five days post-ENT removal. (n=2 donors’ ENT 500nM-treated NK cells) **(C)** CD107a assay using ENT 500nM-treated NK cells. (n=2 donors’ NK cells) **(D)** NK cell survival without IL-2. NK cells were treated with IL-2 and with or without ENT 500nM, then both IL-2 and ENT were withdrawn to confirm their survival. (n=2 donors’ NK cells).

We evaluated the kinetics of CAR expression following ENT removal and resting NK cells overnight. The GFP+ CAR+ population was maintained after resting with reduced CAR MFIs (**Figure 4A**). We further tested the resting effect for five days. GFP+ CAR+ populations continued to decline following ENT withdrawal. Nonetheless, pNK cells maintained higher CAR expression levels five days post-ENT removal than DMSO-treated CAR-pNK cells (**Figure 4B**). To assess the functionality of ENT-treated CAR-IL-15-pNK cells against multiple myeloma cells, we treated cryopreserved NK cells with 500nM ENT. Considering previous observations of a slight reduction in overall MFIs following overnight resting, we also compared degranulation with that of NK cells rested overnight. Treatment with 500nM ENT significantly reduced degranulation in response to CD138-negative Ramos cells and NK cells alone. In contrast, CD107a expression remained mostly intact when targeting CD138-positive MM1.S cells. Notably, allowing NK cells to rest overnight partially restored their function against Ramos cells but slightly diminished degranulation against MM1.S cells. (**Figure 4C**). The slight reduction in degranulation may be attributed to the modest decrease in CAR MFIs post-resting. Therefore, our data suggest that CAR-mediated cytotoxicity remains functional despite ENT treatment.

Next, we evaluated NK cell survival following 500nM ENT treatment. Given that GFP is linked to IL-15 via the P2A sequence under the MSCV promoter, we hypothesized that ENT-treated NK cells with increased GFP expression would demonstrate improved survival without IL-2. NK cells were cultured with 500nM ENT for two days, after which IL-2 and ENT were withdrawn. As hypothesized, non-treated NK cells experienced substantial cell death, with 78% mortality after four days without IL-2. In contrast, ENT-treated NK cells exhibited enhanced survival, with only 39.4% cell death *in vitro* (**Figure 4D**). Altogether, our data demonstrate that ENT treatment enhances transgene expression without compromising CAR-mediated NK cell degranulation.

### ENT treatment of CAR-pNK cells during expansion *ex vivo* upregulates CAR expression and improves target cell lysis

**Figure 5:**
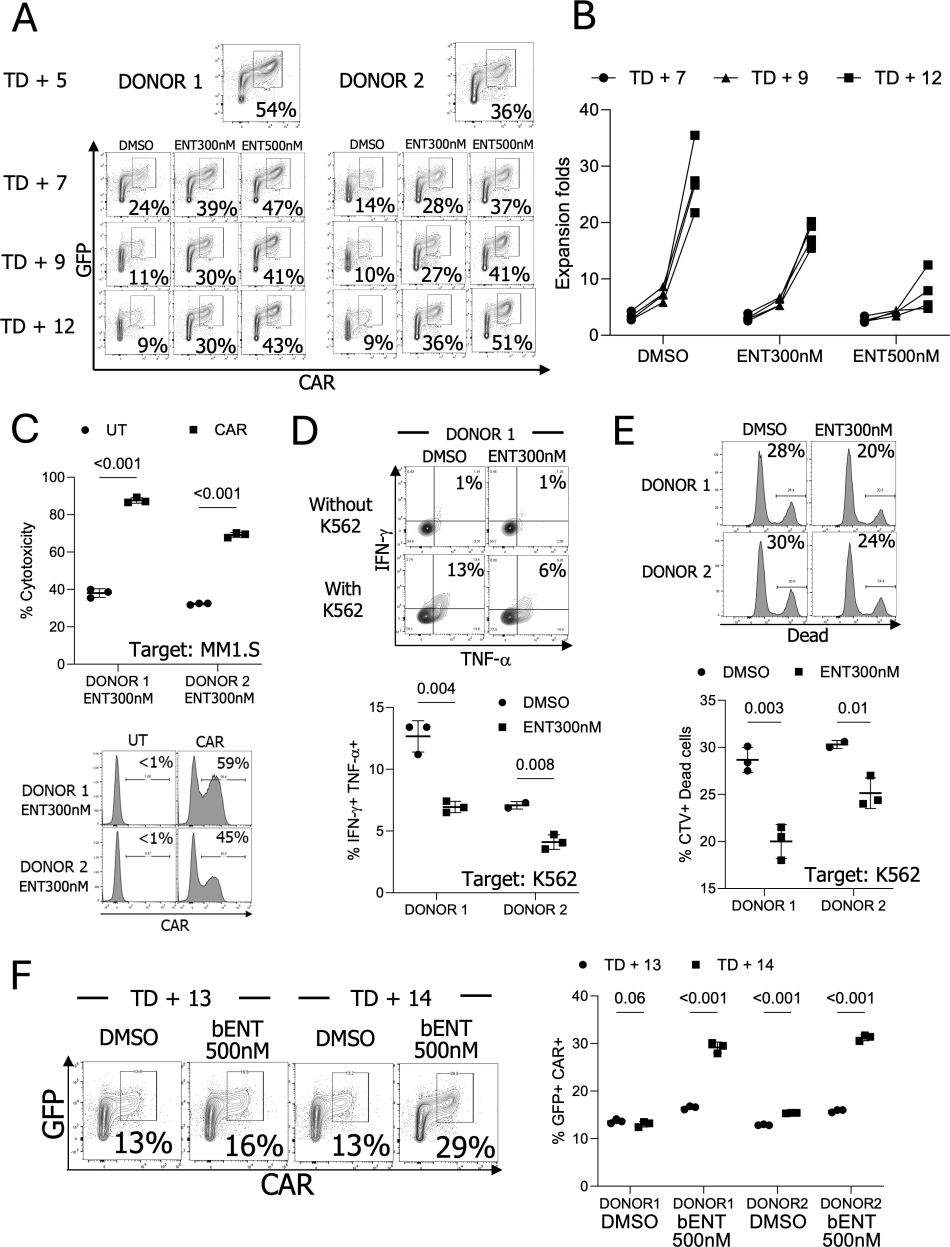
The CAR and NK cell phenotypes after long-term ENT treatment **(A)** CAR expression analysis during expansion *ex vivo* following CAR transduction in the presence of ENT (n=2 donors’ NK cells). **(B)** NK cell expansion for seven days with ENT. (n=4, 2 donors’ NK cells with or without engineering). NK cells were transduced, stimulated, and expanded for five days (TD + 5). The modified NK cells were then cultured with or without ENT. **(C)** Comparison of NK cell cytotoxicity between ENT 300nM-treated CAR NK cells and ENT-treated untransduced (UT) NK cells *in vitro.* (n=2 donors’ NK cells) **(D)** Examine cytokine production in DMSO or ENT 300nM-treated UT-NK cells. (n=2 donors’ NK cells) **(E)** Target cell killing activity of the UT-NK cells. (n=2 donors’ NK cells) **(F)** DMSO-treated TD + 12 NK cells were treated with 500nM ENT for two days (brief stimulation, bENT). NK cells showed improved GFP+ CAR+ populations (n=2 donors’ NK cells).

To assess the possible application of ENT during NK cell expansion, we transduced NK cells and expanded CAR-IL-15-pNK cells for 5 days (TD + 5), followed by treatment with either 300nM or 500nM ENT for 7 days (TD + 12). The 300nM and 500nM were selected due to the CAR maintenance from the HDACi dosage assay. Without ENT, the GFP+ CAR+ NK cell population steadily decreased; however, this population was better maintained in both the 300nM and 500nM ENT treatment groups (**Figure 5A**). NK cells treated with 500nM ENT exhibited reduced expansion, while those treated with 300nM ENT continued to proliferate (17.97-fold for 300nM vs. 7.64-fold for 500nM) (**Figure 5B**).

To confirm NK cell cytotoxicity following long ENT treatment, we incubated both unmodified and CAR-modified NK cells with or without 300nM ENT. Subsequently, 1 × 10^5^ NK cells were co-incubated overnight with 1 × 10^6^ MM1.S-FLUC cells at a 1:10 ratio. The results showed that CAR-expressing NK cells significantly outperformed unmodified NK cells in eradicating target MM1.S cells (**Figure 5C**). Interestingly, Donors 1 and 2, used in this experiment, exhibited comparable cytotoxicity levels without CAR but differed when CAR expression was present. To explore this further, we assessed CAR expression levels in both donors. Donor 1 demonstrated 59% CAR positivity, whereas Donor 2 exhibited 45%, confirming that the enhanced cytotoxicity was predominantly driven by CAR expression (**Figure 5C**). By assessing unmodified NK cells, we verified that this enhanced cytotoxicity was not attributed to ENT treatment. DMSO- or ENT-treated unmodified NK cells were co-incubated with the general NK cell target K562 cells. ENT treatment slightly reduced NK cell cytotoxicity and cytokine production, further supporting that CAR expression is the primary factor of ENT-treated NK cell cytotoxicity (**Figures 5D, E**).

The reduced NK cell proliferation in the presence of ENT during expansion limits its applicability. To overcome this limitation, we assessed whether ENT could increase the GFP+ CAR+ population in NK cells after TD + 12 days. The DMSO-treated TD + 12 NK cells were treated with 500nM ENT (brief treatment, bENT) for two days. Notably, this brief ENT treatment upregulated CAR expression, resulting in high CAR expression (**Figure 5F**). We also validated whether the ENT treatment changes NK cell phenotypes. ENT-treated NK cells showed reduced expression of activating receptors, including NKp30, NKp44, NKp46, and NKG2D (**Supplementary Figure S4**).

### ENT-treated CAR-overexpressing pNK cells show enhanced anti-myeloma activity *in vivo*

**Figure 6:**
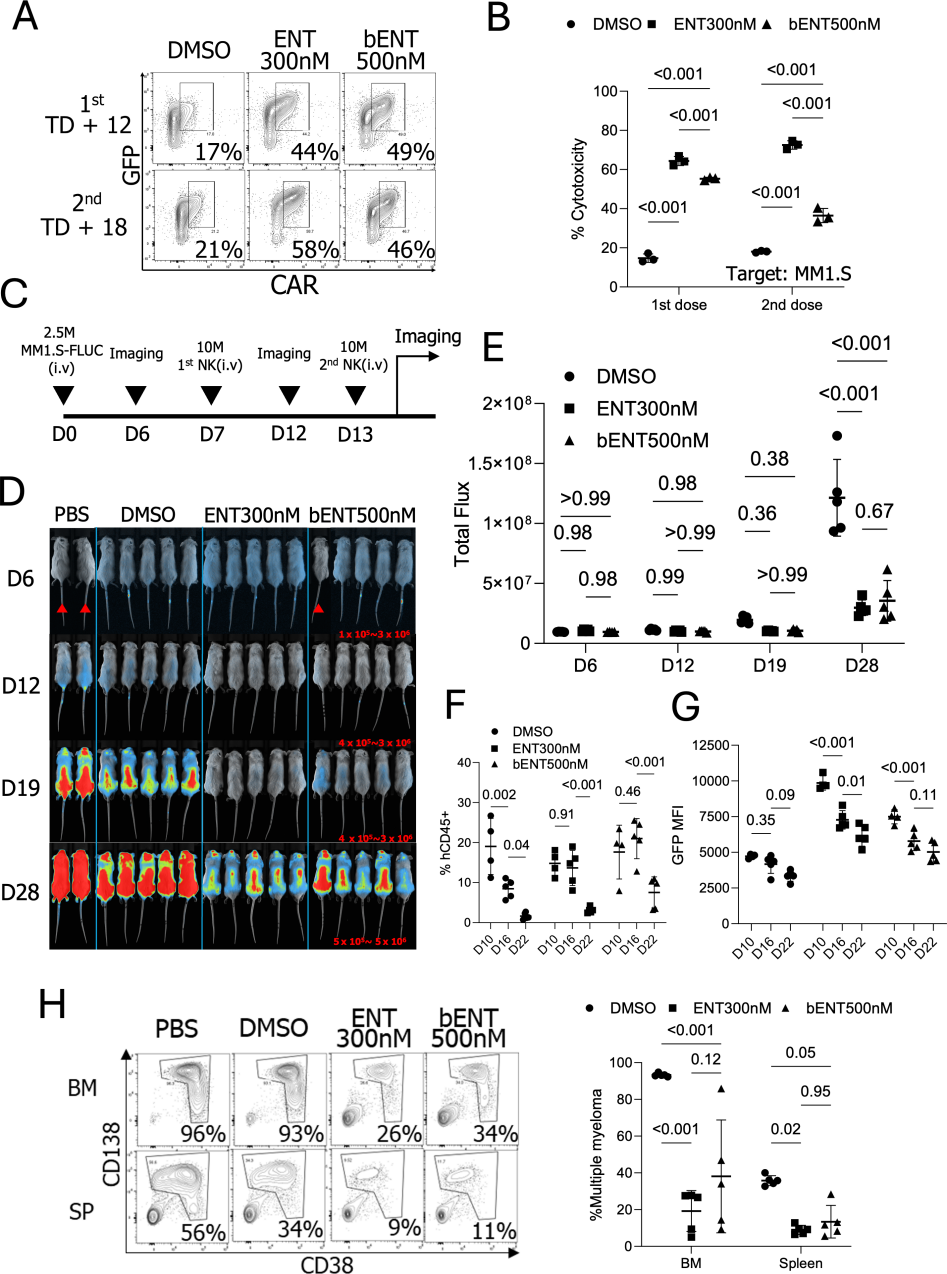
Anti-tumor activity of CAR-NK cells treated with ENT *in vitro* and *in vivo*. **(A)** GFP and CAR expressions in NK cells used for *in vivo* injection. (n=1 donor’s NK cells) **(B)** *In vitro* cytotoxicity of the expanded NK cells. Cytotoxicity was assessed on the day of the NK cell injection. **(C)** Schematic diagram of the *in vivo* experiment. **(D)** Newton imaging. The brighter color (blue to red) indicates the higher luminescence signals from MM1.S-FLUC cells. Marked mice were exposed for 30 seconds and not included in the statistical analysis. Other mice received 2 minutes of exposure. Red numbers indicate the analysis scale for the 2-minute exposed mice. (n=5 mice for NK cell treatments and n=2 mice for PBS control). **(E)** Analysis of total flux from each mouse. **(F)** Percentages of human CD45 positive populations in the blood on days 10, 16, and 22 post-MM1.S-FLUC cell injection. **(G)** GFP MFIs from the hCD45 positive cells. **(H)** Multiple myeloma tumor burden on day 29 in bone marrow (BM) and spleen (SP).; FLUC, Firefly luciferase; bENT 500nM, three days ENT 500nM treated NK cells; BM, Bone marrow. The days in **(D–H)** indicate the timeline post-MM1.S-FLUC cell injection.

To evaluate whether ENT-treated NK cells could improve *in vivo* tumor elimination, we transduced and expanded CAR-pNK cells for 5 days, followed by further expansion with 300nM ENT for 7 days. A bENT group was also included, where CAR-pNK cells were exposed to 500nM ENT for 3 days after TD + 9 days to assess whether short-term treatment could improve tumor control *in vivo*. In the *in vivo* model, 2.5 × 10^6^ MM1.S-FLUC cells were injected intravenously into mice. Based on prior *in vivo* experiments demonstrating that CAR-pNK cells effectively eliminated tumor burdens developed for 3 days, we delayed the first NK cell dose to 7 days post-tumor injection for inducing higher tumor burdens. On the day of NK cell injection (TD + 12), the GFP+ CAR+ populations were 17%, 44%, and 49% for the control, 300nM ENT, and 500nM bENT groups, respectively (**Figure 6A**). Before *in vivo* injection, the cytotoxicity of ENT-treated pNKs was tested *in vitro*. ENT-treated CAR-IL-15-pNK cells demonstrated superior *in vitro* cytotoxicity against MM1.S cells compared to the DMSO-treated pNK cells (**Figure 6B**). After the first dose, the remaining NK cells were further expanded for 6 more days with 300nM ENT or without ENT, followed by another brief stimulation with 500nM ENT. A second dose of CAR-pNK cells was administered to the mice 6 days after the first injection (TD + 18) (**Figure 6C**). After long-term expansion, ENT treatment showed significantly higher GFP+ CAR+ NK cell populations (21%, 58%, and 46% for NT, 300nM ENT, and 500nM bENT, respectively) (**Figure 6A**). *In vivo* imaging data demonstrated that both 300nM and 500nM bENT-treated NK cells provided superior tumor control compared to the DMSO-treated NK cells (**Figures 6D, E**).

Peripheral blood analysis from ENT-treated mice indicated improved NK cell survival. GFP expression in NK cells suggested that ENT treatment temporarily increased GFP-IL-15 gene expression, contributing to short-term NK cell persistence and enhanced tumor control *in vivo* (**Figures 6F, G**). After 29 days of tumor engraftment, mice were euthanized, and tumor burdens in the bone marrow and spleen were analyzed. Consistent with luminescence data, the percentage of multiple myeloma was significantly lower in mice that received 300nM or brief-500nM ENT-treated NK cells (**Figure 6H**). In conclusion, CAR-IL-15-pNK cells treated with ENT during *ex vivo* expansion demonstrated enhanced CAR and IL-15 expression, resulting in superior anti-myeloma activity *in vivo*.

## Discussion

Multiple myeloma is characterized by the uncontrolled proliferation of highly differentiated malignant plasma cells (9). The clonal diversity and differentiation of these plasma cells contribute to high relapse rates in MM patients treated with antibody- or proteasome inhibitor-based therapies (11). This study investigates CAR-NK cells targeting CD138, a marker highly expressed in multiple myeloma cells (14). While anti-CD138 CAR-NK cells effectively eradicated target myeloma cells *in vitro*, CAR surface expression on NK cells declined over time during *ex vivo* NK cell expansion, limiting their therapeutic efficacy. To overcome the progressive loss of CAR expression, we assessed the feasibility of using cryopreserved NK cells engineered with an anti-CD138 CAR, combined with histone deacetylase inhibitors, thereby improving treatment outcomes for multiple myeloma.

One critical component in NK cell research is the expansion process, which can be influenced by factors such as blood donors, irradiated feeder cells, and NK cell isolation methods (39, 40). The importance of the NK cell expansion becomes more significant when NK cells are engineered, for example, with CAR transgenes. In this research, we tested the feasibility of using primary NK cells cryopreserved at early stimulation stages for engineering and subsequently expanding them after a freeze/thaw cycle. We observed that NK cells generally reached the exponential growth phase around 5 days after the first feeder cell stimulation. Cryopreserving NK cells at this stage consistently yielded high survival rates after thawing. Additionally, NK cells, thawed after being stored at -80°C for three months, still exhibited successful CAR engineering and robust expansion with feeder cell stimulation post-transduction. This cryopreservation strategy provides flexibility in CAR-NK cell preparation for preclinical experiments and presents a potential advantage for the clinical application of off-the-shelf CAR-NK cell immunotherapy.

Due to the frontline intracellular epigenetic modification pathways, foreign transgenes are prone to gene silencing in various cell types (16). In particular, CAR downmodulation during *ex vivo* expansion impacts the efficacy of NK cell-mediated immunotherapy. Our study aimed to boost CAR expression during *ex vivo* expansion to support long-term expansion and a multi-dosing strategy. To address this issue of CAR downregulation, we decided to test HDAC inhibitors, which are epigenetic modifiers due to the previously reported papers showing HDACi-induced transgene overexpression (19–22). Remarkably, HDACi treatment was able to restore transgene expression, sustaining CAR expression during *ex vivo* expansion. This sustained expression allowed for more effective and durable CAR-NK cell function, significantly reducing CAR downregulation. Furthermore, HDACi can be applied during the final two days of the *ex vivo* expansion phase to restore CAR expression. Typically, NK cell expansion is limited to 2-3 weeks, as CAR expression often diminishes beyond this period. However, brief treatment of CAR-NK cells with HDACi at the end of expansion may extend this duration to 4-5 weeks, addressing a critical bottleneck in CAR-NK cell production. This extended expansion strategy will improve the availability of CAR-NK cells for repeated dosing, enhancing their off-the-shelf potential for more effective therapeutic outcomes and relapse prevention.

In investigating the promoter-specific effects of ENT, we observed that ENT-mediated CAR overexpression driven by the MSCV and CMV promoters in the lentiviral vector system was significantly higher than the effect in the EFS promoter, highlighting the differential regulation of transgene expression in NK cells based on promoter selection. This promoter effect brings an interesting question. The recent advancements in CAR-NK cell generation, such as targeted gene insertion via AAV vectors, which is considered superior to lentiviral systems for CAR expression, often utilize the MND promoter (myeloproliferative sarcoma virus enhancer, negative control region deleted, dl587rev primer-binding site substituted promoter) in NK cells (41). Given that our data indicate varied CAR expressions and differential responses to epigenetic inhibitors across different promoters in NK cells, it would be worthwhile to investigate whether the MND promoter can achieve high CAR expression in NK cells with a lentiviral system and respond to HDACi.

In conclusion, our study demonstrates that applying HDAC inhibitors, especially ENT, can effectively upregulate CAR transgene in a promoter-specific manner, showing sustained CAR and IL-15 transgene expression in NK cells during *ex vivo* expansion. In the presence of ENT, CAR expression remained stable, allowing for prolonged expansion and supporting the feasibility of multi-dosing CAR-NK cells. This combinational approach of CAR-NK cells with ENT will offer significant therapeutic potential for advancing off-the-shelf CAR-NK cell immunotherapy.

## Data Availability

The original contributions presented in the study are included in the article/**Supplementary Material**. Further inquiries can be directed to the corresponding author.
