# Supplementary material for: Entinostat, a histone deacetylase inhibitor, enhances CAR-NK cell anti-tumor activity by sustaining CAR expression
Source: Front Immunol. 2025 Mar 7;16:1533044. doi: 10.3389/fimmu.2025.1533044 (PMC11925867; doi:10.3389/fimmu.2025.1533044)
Supplement: Supplementary file 1 [file DataSheet1.pdf]

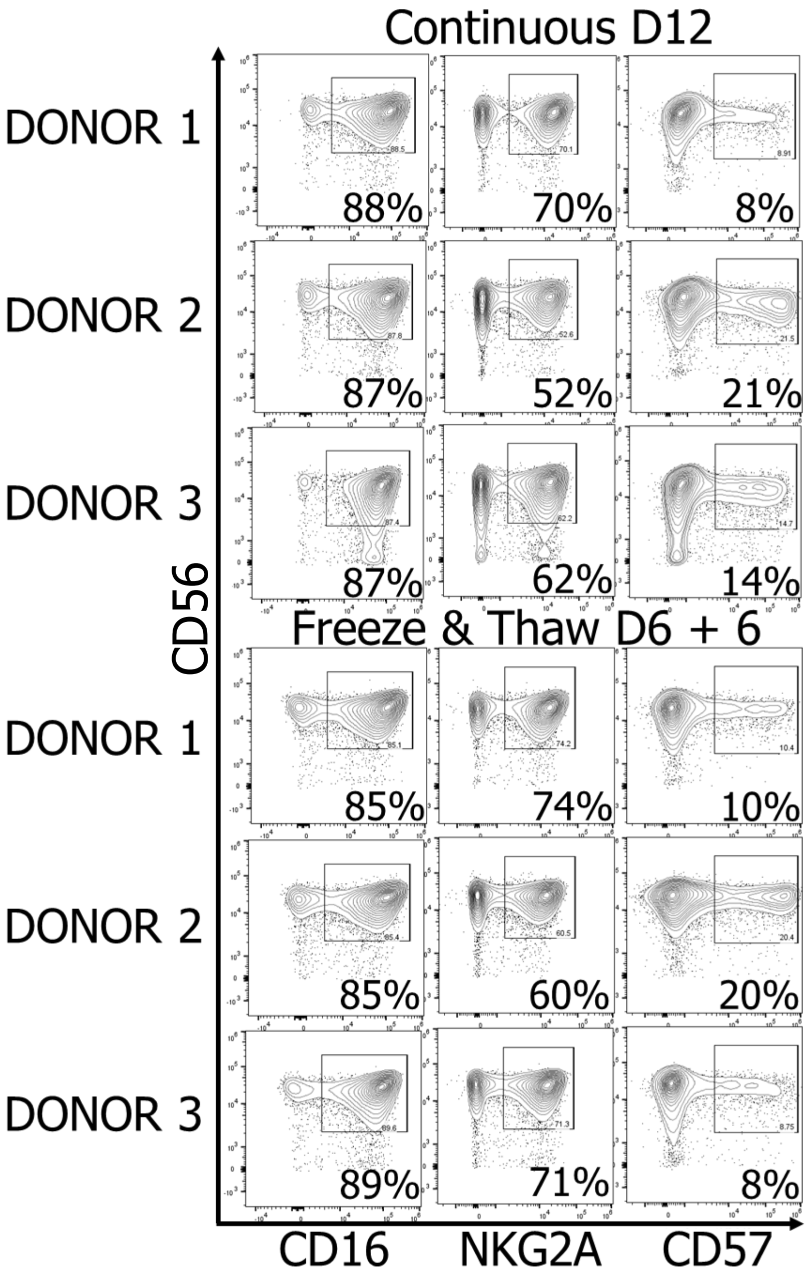

Figure S1. NK cell population markers in continuously expanded and early cryo-preserved-expanded NK cells. Human primary NK cells were expanded over a period of 12 days. Cryo-preservation was performed on day six of the expansion process. NK cells from three different donors (n=3) were used for analysis.

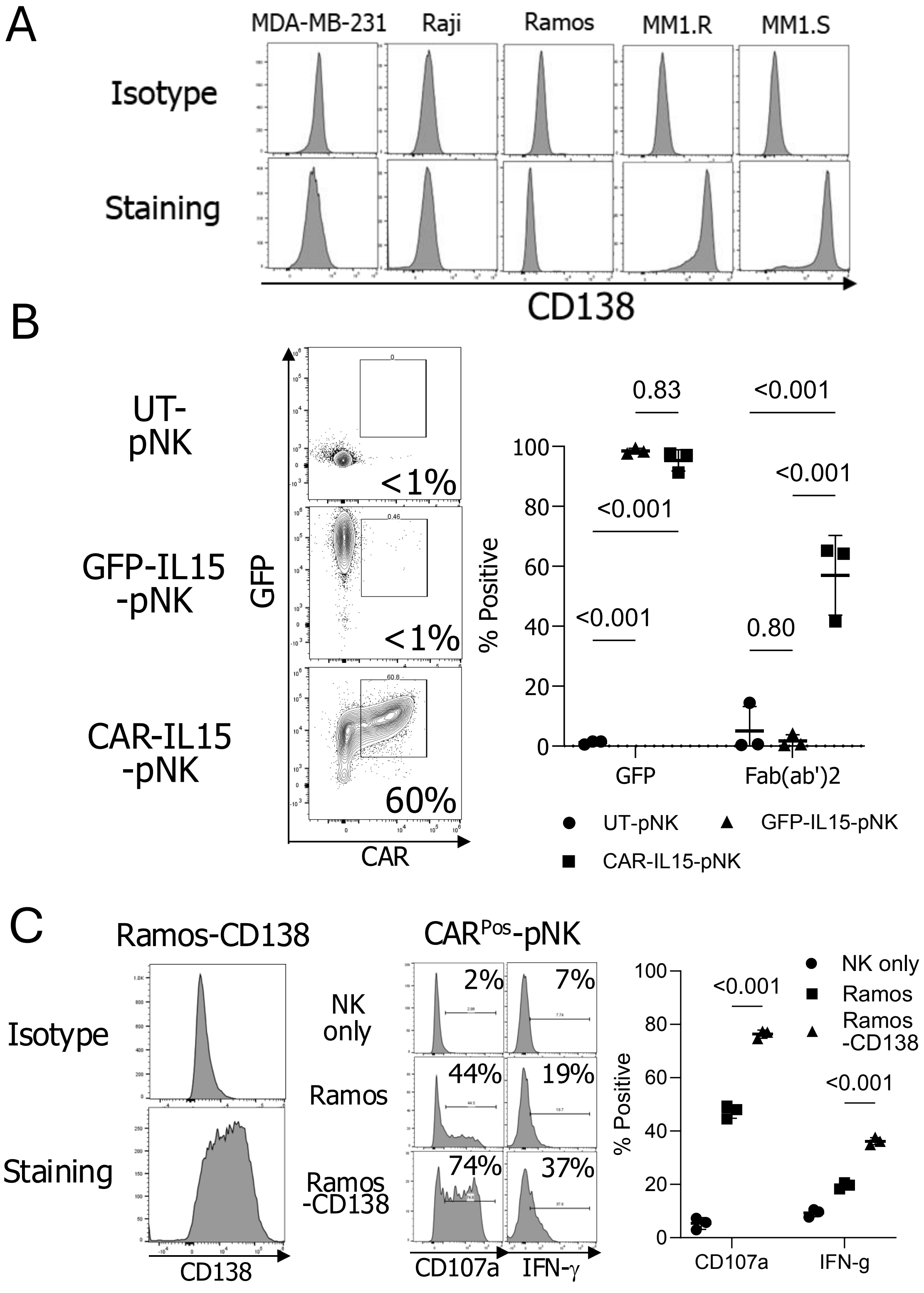

Figure S2. CD138 expression in target cells and pNK cell CAR functionality. (A) CD138 expression in the target cells used in this study was confirmed by flow cytometry. **(B)** GFP and CAR expressions in modified pNK cells. (n=3 cryopreserved donors' NK cells) **(C)** CAR activity confirmation using CD138 expressing Ramos cells.

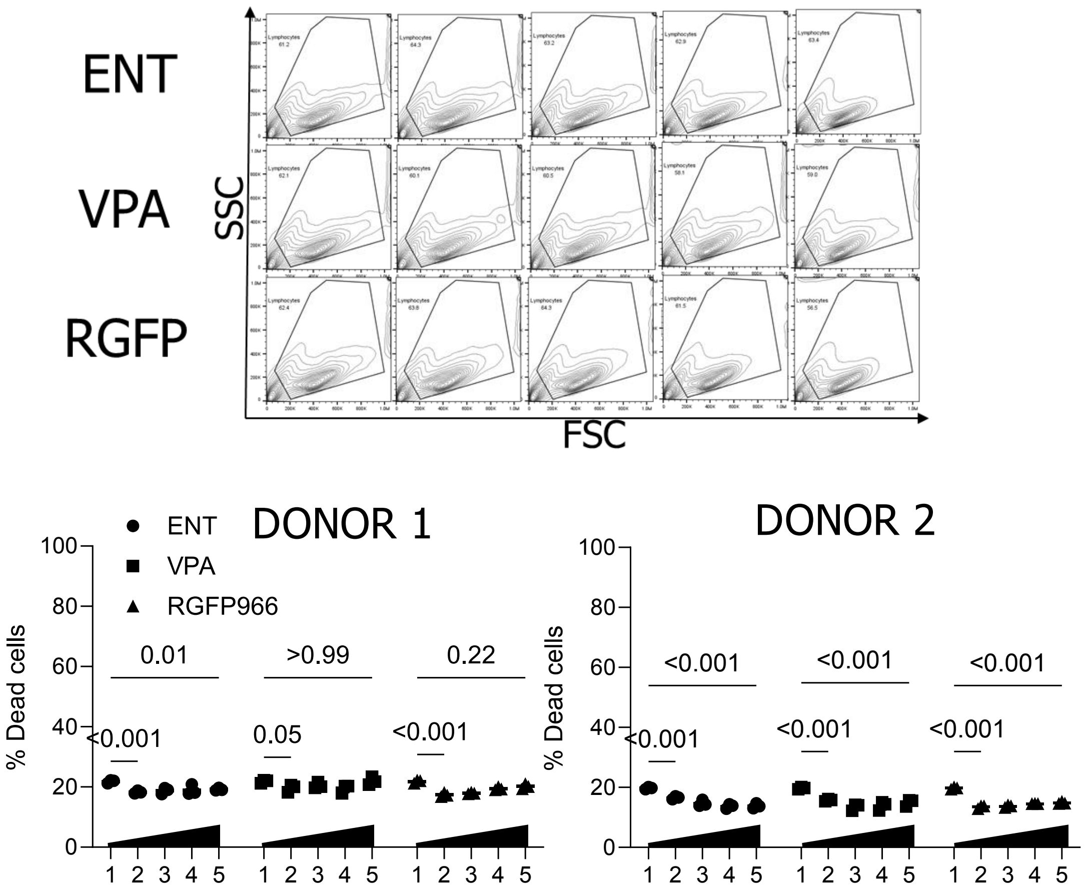

Figure S3. Histone deacetylase inhibitor (HDACi) toxicity. HDACi toxicity was assessed using flow cytometry, with dead cells identified in gated areas. Cells were exposed to various concentrations of inhibitors for two days. The concentrations tested were as follows: ENT (Entinostat): 0 μM, 0.1 μM, 0.3 μM, 0.5 μM, and 1 μM; VPA (Valproic acid): 0 mM, 0.1 mM, 0.3 mM, 0.5 mM, and 1 mM; RGFP (RGFP966): 0 μM, 2.5 μM, 5 μM, 10 μM, and 20 μM.

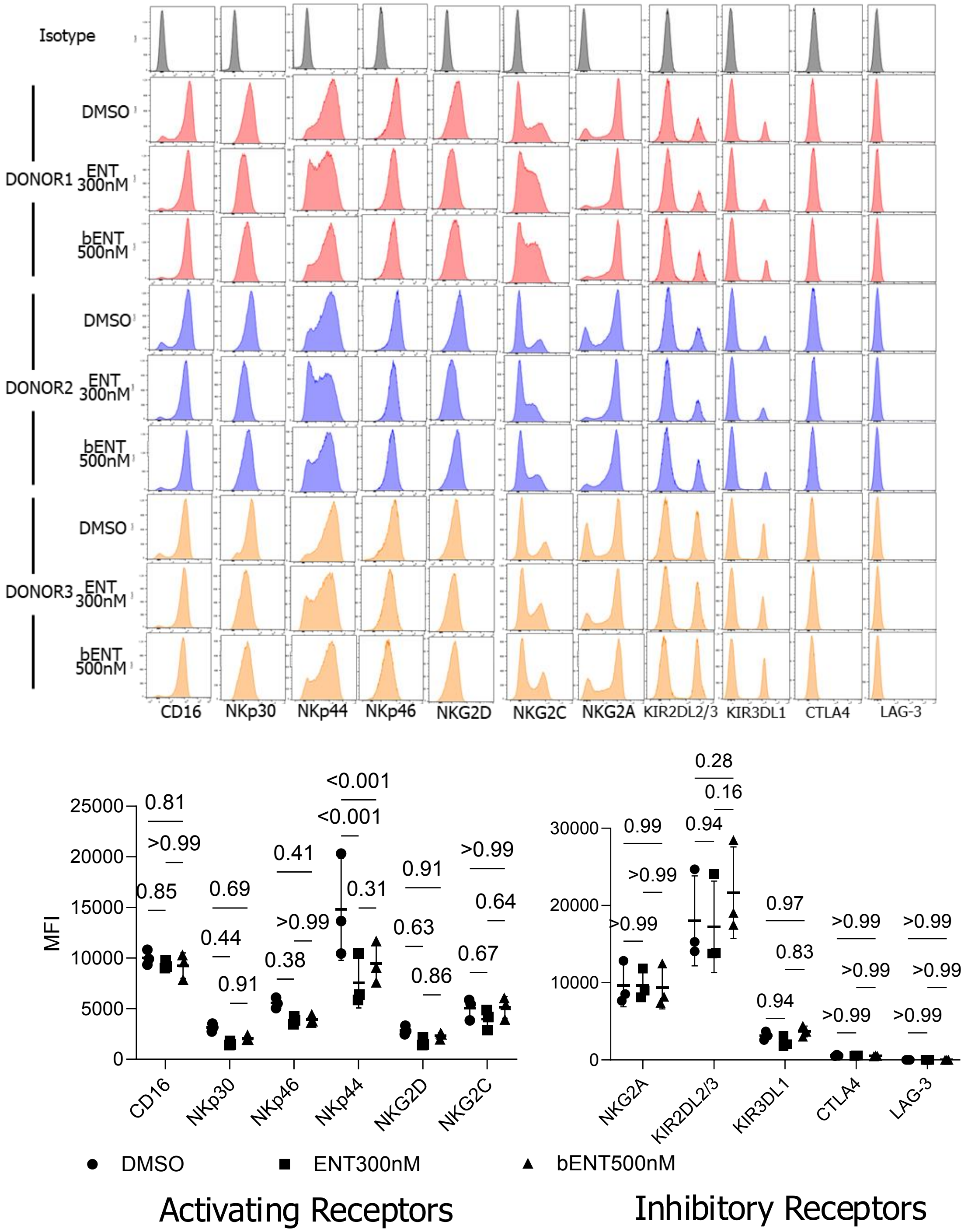

Figure S4. Phenotypes of ENT-treated NK cells. NK cells were modified and expanded for five days (TD + 5), followed by further expansion for an additional seven days, either with or without 300 nM ENT. On TD + 9, DMSO-treated NK cells were divided into two conditions: DMSO or 500 nM ENT (bENT500nM). On TD + 12, NK cells were phenotyped by immunostaining.
